# Ventriculopleural shunt dysfunction as the first sign of a hidden aneurysmal Subarachnoid Hemorrhage: A case report

**DOI:** 10.1016/j.amsu.2020.08.018

**Published:** 2020-08-21

**Authors:** Daniel Alejandro Vega-Moreno, María Elena Córdoba-Mosqueda, José Ramón Aguilar-Calderón, Rodrigo Efraín Hernández-Resendiz, Heberseleth Valdivia-Chiñas, Erick Alberto Castañeda-Ramírez, Óscar Medina-Carrillo, Rafael Sánchez-Mata

**Affiliations:** a Neurosurgery Department, Hospital Central Sur de Alta Especialidad, PEMEX, Periférico sur 4091, Fuentes del Pedregal, Tlalpan, 14140, Mexico City, Mexico; b Neurosurgery Department, Hospital Ángeles Clínica Londres, Frontera 74, Roma Norte, Cuahutémoc, 06700, Mexico City, Mexico; c Neurosurgery Department, Hospital General Zona 33, Av. Félix U. Gómez, Centro, 64010, Monterrey, Nuevo León, Mexico; d Neurosurgery Department, Hospital General Regional No1 IMSS, Ciudad Obregón, Morelos, 85110, Sonora, Mexico

**Keywords:** Subarachnoid hemorrhage, Cerebral aneurysms, Shunt dysfunction, Lumbar puncture, Case report, SAH, Subarachnoid Hemorrhage, CT, Computed Tomography, LP, Lumbar Puncture, CSF, Cerebrospinal Fluid

## Abstract

**Introduction:**

Subarachnoid Hemorrhage (SAH) is caused by an aneurysmatic origin in 80% of cases. In the adult population, the risk of shunt dysfunction is about 16% in the first year, with proximal mechanical obstruction being the most frequent cause.

**Case report:**

An 81-year-old man with a history of shunt system placement presented among clinical data of shunt dysfunction. The brain Computed Tomography (CT) showed dilation of the ventricular system, with no other associated injury. The cause of the dysfunction was a SAH determined by a lumbar puncture (LP) study. We performed an angiography reporting 3 aneurysms.

**Discussion:**

The risk of shunt dysfunction at one year is 40% and at two years, the risk ups to 53% with obstruction of the system and infection being the two principal causes. The usefulness of a lumbar puncture for late detection of SAH lies in the red cells in the Cerebrospinal Fluid (CSF). When the CT is negative and the clinical suspicion remains, the lumbar puncture (LP) continues with higher sensitivity despite is over 12 hours of the onset clinic symptoms.

**Conclusion:**

This case encourages to follow a rigorous protocol study for patients with multiple shunt dysfunction and chronic hydrocephalus. Also, this case invites to consider a hidden SAH secondary to a vascular pathology as a differential diagnosis for a multiple shunt dysfunction.

## Highlights

• The principal causes of shunt dysfunction are obstruction and infection.
• Lumbar puncture in the firsts days has 100% sensitivity for Subarachnoid Hemorrhage.
• Negative image for Subarachnoid Hemorrhage can be positive in Lumbar Puncture.

## 1 Introduction

The cause of a Subarachnoid Hemorrhage (SAH) in 80% of cases is because of an aneurysmatic origin with a lethality range from 25% to 50%. The global incidence of cerebral aneurysms is 1%–2% [1,2], without differences between Caucasian, African or Latin American population [3].

Clinical presentation of SAH is a sudden and severe headache, referred as the worst in life, sometimes preceded by a sentinel headache in up to 40% of patients, weeks before the acute condition. Nausea, vomiting, stiffness, loss of consciousness or focal neurological deficit may appear in varying degrees of presentation [1].

Obstruction, infection of the valve, catheter disconnection and malpositioning of the ventricular or peritoneal catheter are the most frequent causes of shunt malfunctions. In the pediatric population, the range of complications is between 45% and 59%, and about 16% in the first year in the adult population [4].

This investigation aims to describe the approach of a patient with a shunt dysfunction as the first sign of hidden SAH secondary to an aneurysm rupture and chronic hydrocephalus. This work has been reported in line with the SCARE criteria [5].

## 2 Case report

In 2005, an 81-year-old man with a medical history of smoking, systemic hypertension, and type 2 diabetes mellitus, presented SAH with ventricular irruption secondary to traumatic etiology, treated with a ventriculoperitoneal shunt. After 3, 12, and 24 months, he required shunt replacements for an unspecified etiology, the last was a ventriculopleural shunt.

The patient sought medical attention through his primary care physician complaining of one-month history of attention disturbances, behavior changes, instability and urinary incontinence. With the clinical impression of a new shunt disfunction, the physician referred the patient to the neurosurgery department of our hospital for evaluation and treatment.

When the patient arrived to admission, he was inattentive and disoriented, with a Glasgow Coma Score of 14. He presented bilateral papilledema, wide-based gait in brief steps, negative Kerning, Brudzinski, and Binda signs and absent of neck stiffness. The shunt reservoir did not expand after applying pressure, showing a proximal obstruction.

**Fig. 1:**
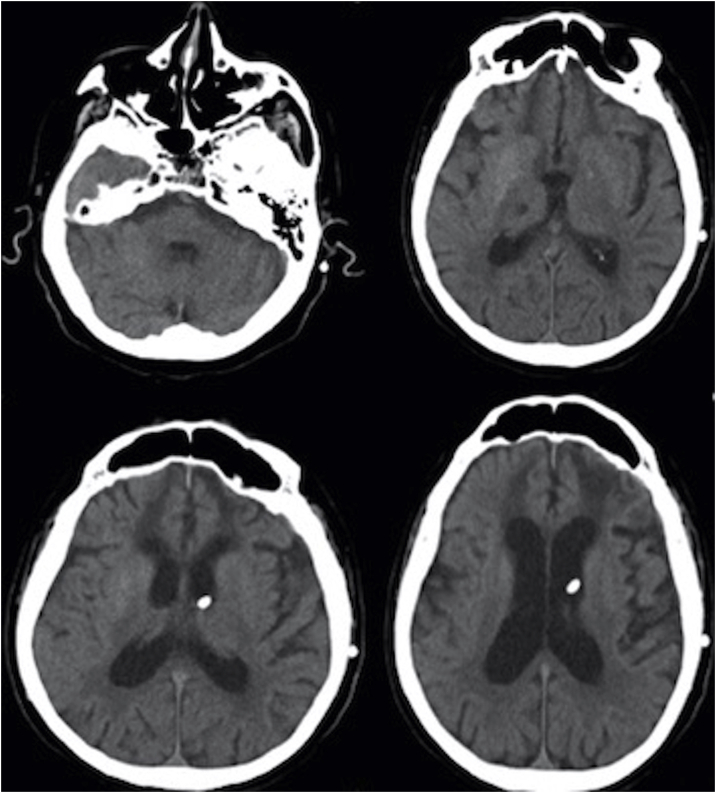
Cerebral Computed Tomography, axial view. Dilation of the ventricular system. Interstitial periventricular edema. Absence of blood density in the ventricular system.

We performed blood, and urinary tests obtaining normal results. The CT reported dilation of the ventricular system and interstitial periventricular edema (Fig. 1). After these results we realized a LP showing: xanthochromic, 1292 cells at the expense of crenocytes (885) and erythrocytes (405), glucose 114 mg/deciliter; stains and cultures were negative.

**Fig. 2:**
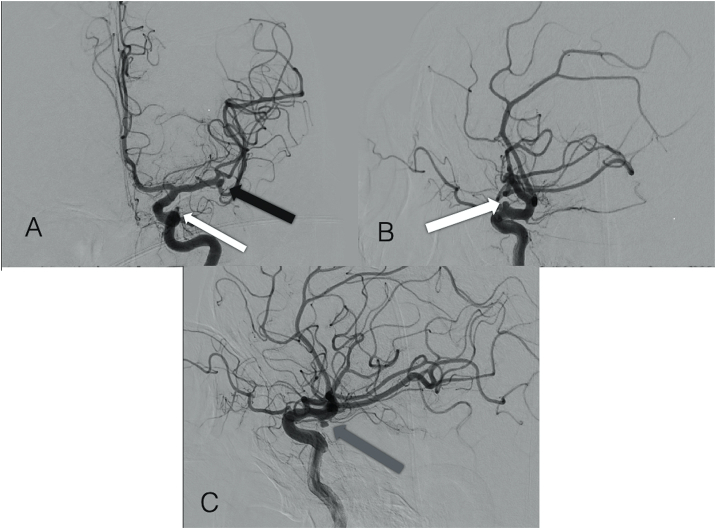
Angiography. A) Anteroposterior view, and B) lateral view showing left Internal Carotid with a small saccular aneurysm in the left ophthalmic segment (white arrow) and a saccular aneurysm in the left Medial Cerebral Artery at M2 segment (black arrow). C) Lateral view showing right Internal Carotid with a blister aneurysm in the ophthalmic segment (gray arrow).

Based on the LP results, we established the diagnosis of Subarachnoid Hemorrhage (SAH). The results showed that three aneurysms were identified on the angiography, a small saccular aneurysm in the left medial cerebral artery at M2 segment, a blister aneurysm in the left ophthalmic segment of the internal carotid artery (C6) and a small saccular aneurysm in the right ophthalmic segment of the internal carotid artery (C6) (Fig. 2).

We performed a shunt replacement of the proximal catheter. After two days of follow-up, we discharged the patient with improved gait, attention, and behavior. We referred him to the neurovascular department to treat the aneurysmatic disease.

## 3 Discussion

The predominant symptoms of shunt dysfunction are headache, nausea, vomiting, gait disturbances, visual alterations, neuropsychological deficits, and urinal incontinence [6]. The risk of shunt dysfunction according to the international literature is from 30% to 50% after 3 years follow-up [4]. The clinical presentation of our patient led us to suspect a new shunt dysfunction and according to the study protocol reported in the international literature we realized a CT scan and LP to find the etiology [6].

The predominant symptom in Subarachnoid Hemorrhage (SAH) is a headache classically described as a “thunderclap headache”; however, to determine the etiology the first study needed is CT [7]. The sensitivity of the CT for SAH reaches a 100% the first three days and decreases to 50% one week after the onset of the symptoms: 0.4% of negative CTs for SAH can be positive in LP [1].

In our case, the CT scan did not show any etiology because our patient sought medical attention four weeks after the onset of his symptoms, decreasing the CT sensitivity; however, the CSF findings led us to consider SAH as the origin of shunt dysfunction.

The usefulness of a lumbar puncture for the late detection of SAH lies in the red blood cells in the Cerebrospinal Fluid (CSF), undergo lysis and phagocytosis: this releases oxyhemoglobin and converted into bilirubin.

When the CT is negative and the clinical suspicion remains, we base the diagnosis on CSF analysis for red blood cells, xanthochromia and bilirubin [8]. This data differentiates between a traumatic puncture and bleeding in the ventricular system.

After bleeding, the sensitivity in the first 15 days is around 100%, 70% in the following 3 weeks, and 40% in the following 4 weeks [8]. Despite the fact that our patient didn't receive medical attention in time, LP continues to be more sensitive than CT for SAH, as we present.

Blister aneurysms are rare vascular lesions. The most common localization of these aneurysms is supraclinoid with a proportion of 0.3%–1.7% of all aneurysms and up to 6.6% of aneurysmal ruptures [9]. Blister aneurysms have a higher risk of complications, such as rebleeding or even mortality [10]. For the other aneurysms, we could continue a follow-up, considering the age and comorbidities of the patient.

## 4 Conclusions

When a patient presents a history of multiple shunt dysfunction, it's necessary to apply a complete diagnostic protocol with a CT and LP, as well as a full clinical workup.

When we have a negative result of the CT for SAH, but CSF with positive findings, the following step must be an angiography to find the vascular etiology, since this study is the gold standard to detect multiple aneurysms and other vascular pathologies.

This case encourages to follow a rigorous protocol study for patients with multiple shunt dysfunction and chronic hydrocephalus. Also, this case invites to consider a hidden SAH secondary to a vascular pathology as a differential diagnosis for a multiple shunt dysfunction in an older patient.

## Funding sources

This research did not receive any specific grant from funding agencies in the public, commercial, or not-for-profit sectors.

## Consent

Written informed consent was obtained from the patient for publication of this case report and accompanying images. A copy of the written consent is available for review by the Editor-in-Chief of this journal on request.

## Provenance and peer review

Not commissioned, externally peer reviewed.

## Ethical approval

This article does not warrant approval by the ethics committee.

## Author contribution

Daniel Alejandro Vega-Moreno: Term, Writing-Original draft.

Maria Elena Córdoba-Mosqueda: Writing-Review & Editing, Resources.

José Ramón Aguilar-Calderón: Supervision.

Rodrigo Efraín Hernandez-Resendiz: Conceptualization.

Heberseleth Valdivia-Chiñas: Analysis.

Erick Alberto Castañeda-Ramirez: Important intellectual content.

Oscar Medina-Carrillo: Project administration.

Rafael Sánchez-Mata: Investigation.

## Guarantor

Daniel Alejandro Vega-Moreno.

Maria Elena Cordoba-Mosqueda.

## Declaration of competing interest

None of the participating authors has conflicts of interest or any type of sponsorship.
